# The bile salt glycocholate induces global changes in gene and protein expression and activates virulence in enterotoxigenic *Escherichia coli*

**DOI:** 10.1038/s41598-018-36414-z

**Published:** 2019-01-14

**Authors:** Enrique Joffre, Matilda Nicklasson, Sandra Álvarez-Carretero, Xue Xiao, Lei Sun, Intawat Nookaew, Baoli Zhu, Åsa Sjöling

**Affiliations:** 1 Department of Microbiology, Tumor and Cell biology, Karolinska Institutet, Stockholm, Box 280, S-17177 Stockholm, Sweden; 2 Institute of Biomedicine, Department of Microbiology and Immunology, University of Gothenburg, Box 435, S-405 30 Göteborg, Sweden; 3 CAS Key Laboratory of Pathogenic Microbiology & Immunology, Institute of Microbiology, Chinese Academy of Sciences, Beijing, China; 4 Department of Biomedical Informatics, College of Medicine, The University of Arkansas for Medical Sciences, Little Rock, AR 72205 USA; 5 Collaborative Innovation Center for Diagnosis and Treatment of Infectious Diseases, The First Affiliated Hospital, College of Medicine, Zhejiang University, Hangzhou, China; 6 Department of Pathogenic Biology, School of Basic Medical Sciences, Southwest Medical University, Zhongshan Road, Luzhou, Sichuan China

## Abstract

Pathogenic bacteria use specific host factors to modulate virulence and stress responses during infection. We found previously that the host factor bile and the bile component glyco-conjugated cholate (NaGCH, sodium glycocholate) upregulate the colonization factor CS5 in enterotoxigenic *Escherichia coli* (ETEC). To further understand the global regulatory effects of bile and NaGCH, we performed Illumina RNA-Seq and found that crude bile and NaGCH altered the expression of 61 genes in CS5 + CS6 ETEC isolates. The most striking finding was high induction of the CS5 operon (*csfA-F*), its putative transcription factor *csvR*, and the putative ETEC virulence factor *cexE*. iTRAQ-coupled LC-MS/MS proteomic analyses verified induction of the plasmid-borne virulence proteins CS5 and CexE and also showed that NaGCH affected the expression of bacterial membrane proteins. Furthermore, NaGCH induced bacteria to aggregate, increased their adherence to epithelial cells, and reduced their motility. Our results indicate that CS5 + CS6 ETEC use NaGCH present in the small intestine as a signal to initiate colonization of the epithelium.

## Introduction

Enterotoxigenic *Escherichia coli* (ETEC) is one of the most common bacterial causes of acute watery diarrhea in the developing world, both among children under five years old and in indigenous adults or travelers to endemic regions^1^. ETEC also causes food-borne outbreaks of gastroenteritis in the US and Europe and is often spread by contaminated food and water^2,3^. ETEC colonizes the small intestine, where diarrhea is caused by at least one of two enterotoxins, a heat-stable toxin (ST, including the human STh and human/porcine STp subtypes) or a heat-labile toxin (LT)^4^. Adhesion to the epithelium of the small intestine is mediated by colonization factors (CFs), a heterogeneous group of fimbrial, fibrillar, and afimbrial structures on the bacterial surface. Thus, far up to 27 CFs have been described and verified^5–11^; these may be expressed alone or in different combinations depending on the ETEC strain^4^. Both the enterotoxins and CFs are encoded on plasmids^12^.

Upon entering the host, ETEC bacteria must pass through the acidic environment of the stomach; overcome the bactericidal effects of bile in the duodenum, jejunum, and ileum; and traverse the mucous layer of the small intestine before colonizing the epithelial surface and establishing an infection. Several enteropathogenic bacteria are known to respond to host factors and to alter the expression of virulence factors in response to conditions in the gastrointestinal tract (e.g., pH, bile, bicarbonate, mucus, alkalinity, and high osmolarity)^13–21^. Thus, they are able to adjust their degree of virulence to the most favorable niche of infection.

An important host factor is bile, which is produced by the liver, stored in the gallbladder, and secreted into the duodenum. Bile is composed primarily of un-conjugated and glyco- or tauro-conjugated bile salts, as well as cholesterol, phospholipids, and biliverdin^13,14^. The concentration of bile salt in the duodenum varies between 0.2% and 2%^15^. Bile and some of its individual components, such as the major primary bile salt cholate and the secondary bile salt deoxycholate, as well as the conjugated primary salts taurocholate and glycocholate^16^, are known inducers of virulence in bacterial enteropathogens such as ETEC, *V. cholerae*, enteropathogenic *E. coli* (EPEC), *Campylobacter jejuni*, and *Shigella* spp^17–22^. Because it is a detergent, bile is also involved in the host antimicrobial defense, making it one of the most intriguing host signals affecting virulence. To colonize the human intestine, enteric bacteria such as *E. coli* and *V. cholerae* overcome bile toxicity by regulating outer membrane porins and efflux systems, including *ompU* and *acrAB*-*tolC*, to induce bile resistance^23,24^.

Five ETEC CFs are only expressed on the bacterial surface when bile is present^25^. One such bile-induced colonization factor is coli surface antigen 5 (CS5), one of the most common CFs detected worldwide^26^. CS5 is a fibrillar antigen, and its operon encodes a major subunit (*csfA*), a minor subunit (*csfD*), an outer membrane usher (*csfC*), two chaperones (*csfB* and *csfF*), and a protein involved in regulating pilus length (*csfE*)^27^. ETEC strains expressing CS5 also co-express the operon encoding coli surface antigen 6 (CS6)^6,26^. We showed recently that two individual bile salts, sodium glycocholate (NaGCH) and sodium deoxycholate (NaDC), are each sufficient to induce the expression of CS5 *in vitro*^17^. NaGCH is the sodium salt of the glyco-conjugated primary bile cholic acid and is one of the main components of bile^28^. In contrast to NaDC, NaGCH had previously not been reported to induce virulence among enteropathogenic bacteria^18,20,29^. The goal of the present study was to further explore the contribution of bile, and NaGCH in particular, to global gene and protein expression in ETEC strains expressing CS5 and CS6. To this end, we employed a whole- transcriptome and -proteome approach using RNA sequencing and iTRAQ-coupled LC-MS/MS technology.

## Results

### Bile and the major bile component NaGCH modulate the ETEC transcriptome during mid-logarithmic growth

**Figure 1:**
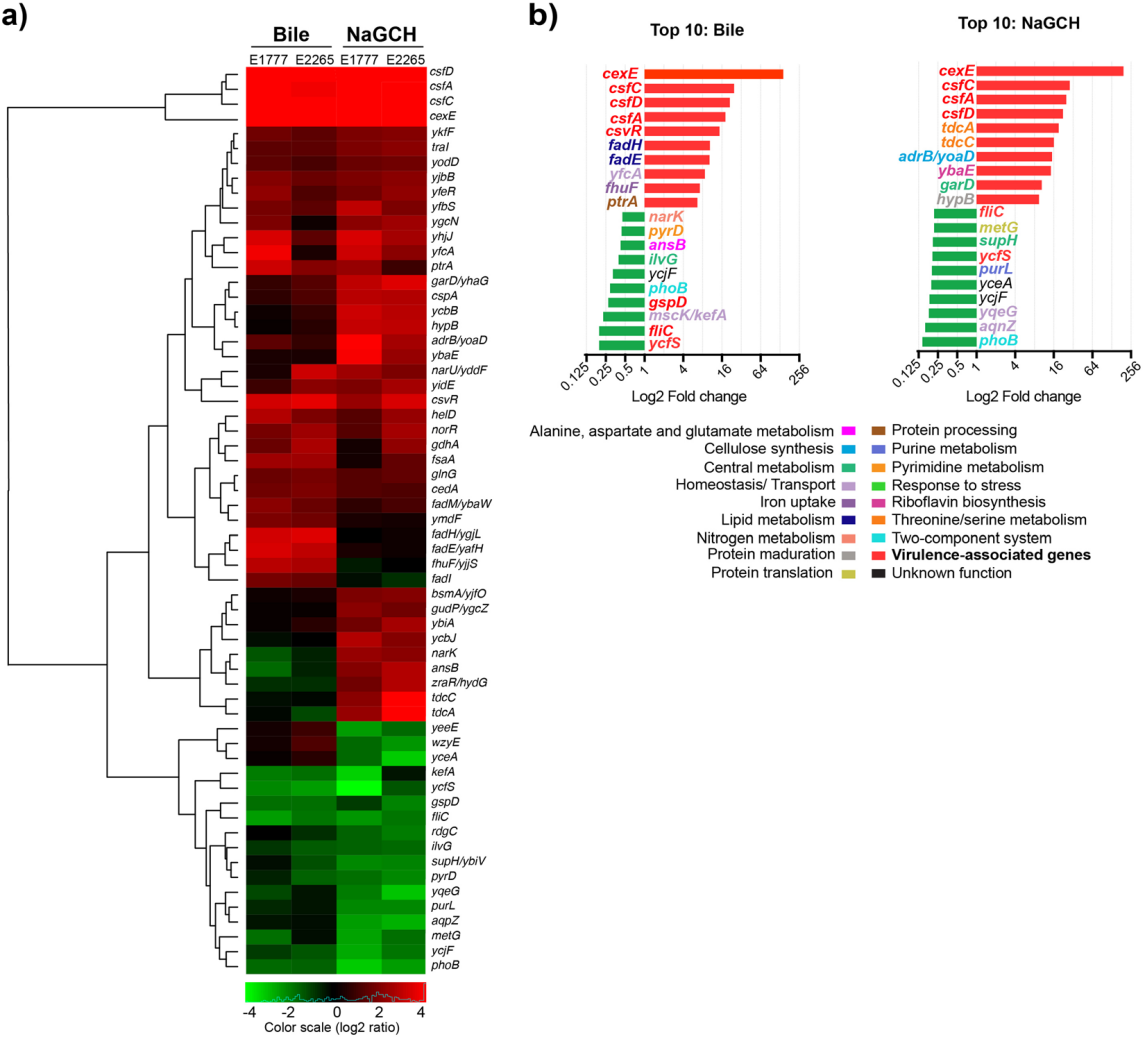
Modulation of ETEC transcriptome in the presence of bile or NaGCH. (**a**) Heat map and hierarchical clustering of 61 genes differentially expressed (>3-fold change) in the presence of bile or NaGCH in the CS5 + CS6 ETEC strains E1777 and E2265. Color scale represents relative expression levels: green indicates low level (downregulated genes), red indicates high level (upregulated genes), and black indicates no change. (**b**) The 10 genes most significantly upregulated (red) or downregulated (green) by bile or NaGCH, including KEGG pathways of the corresponding genes. Values are the average fold change for E1777 and E2265.

Two ETEC clinical isolates, E1777 and E2265, each expressing the virulence factors LT, STh, CS5, and CS6 were analyzed by RNA-Seq for their transcriptional response to bile (0.15%) and the bile component NaGCH (0.2%). Genes up- or down-regulated at least three-fold relative to LB medium were analyzed further; isolate-specific differences in gene expression were excluded to only consider changes in both isolates. With these criteria, we identified 61 genes that were differentially expressed in LB versus bile or in LB versus NaGCH (Fig. 1a, Supplementary Table S1). Among these 61 genes, we found 3 expression patterns: 35 genes were induced by bile and/or NaGCH; 17 genes were repressed by bile and/or NaGCH; and 9 genes were not affected or were downregulated by bile, but upregulated by NaGCH (Fig. 1a).

### Bile and NaGCH affect the expression of genes involved in fatty acid degradation and threonine/serine metabolism

To further explore the effects of bile and NaGCH, we searched the KEGG DATABASE for the differentially expressed genes identified with RNA-Seq. Bile, but not NaGCH, upregulated genes for fatty acid degradation, including *fadE* and *fadI* (for fatty acid β-oxidation), *fadM* (for oleate β-oxidation), and *fadH* (intermediate reductase) (Fig. 1a and b, Supplementary Table S1). Conversely, NaGCH affected genes involved in the transport and metabolism of threonine and serine. NaGCH, but not bile, upregulated *tdcA* (transcriptional activator of the *tdcABC* operon) and *tdcC* (the threonine-serine permease); these effects were especially prominent in E2265, with more than a 25-fold change in gene expression (Fig. 1b).

Several of the changes in gene expression were more pronounced in the presence of NaGCH than bile, only reaching significance with NaGCH. Because NaGCH is a fraction of the crude bile mix, these genes might respond specifically to NaGCH in a dose-dependent manner. For example, *bsmA*, *yodD* (both biofilm-related), and *adrB/yoaD* (for cellulose synthesis) were significantly upregulated by NaGCH, but less so by bile (Fig. 1). Several genes involved in nitrogen metabolism, *narU*, *norR*, and *glnG*, were upregulated by both NaGCH and bile, but *narK* was only upregulated by NaGCH (Fig. 1, Supplementary Table S2). These results indicate that the complex mix of components in bile might mask the specific action of NaGCH. However, it is clear that NaGCH induces significant changes in gene expression in ETEC.

### Bile and NaGCH induce ETEC-specific virulence factors but downregulate flagella genes and PhoB

The genes most significantly upregulated by bile and NaGCH were virulence genes (Fig. 1b). Consistent with our previous results^17^, *csfA* (CS5 major subunit), *csfD* (CS5 minor subunit), and *csfC* (outer membrane usher), all members of the CS5 operon, were highly induced by both bile and NaGCH. Likewise, bile and NaGCH upregulated *csvR*, a member of the AraC/XylS family of transcriptional regulators, and likely the positive regulator of CS5^6^. However, the most abundant transcript found in E1777 and E2265 strains in the presence of either bile or NaGCH corresponded to a variant of the plasmid-encoded gene *cexE* (cfaD-dependent expression, extracytoplasmic protein) (Fig. 1b). CexE was described recently as a putative ETEC virulence factor^30^.

Two genes associated directly with ETEC pathogenesis, *gspD* and *ycfS*, were significantly downregulated in bile and tended toward downregulation in NaGCH; *gspD* is part of the type-2 secretion system involved in LT secretion, and *ycfS* is involved in flagella biogenesis and motility (Fig. 1a and b). Finally, the transcription factor *phoB*, part of the two-component system that positively regulates the phosphate regulon, and the gene *fliC* that encodes the flagellin, were strongly downregulated by both bile and NaGCH (Fig. 1b, Supplementary Table S1).

### Effects of NaGCH on the quantitative proteomic profile of CS5 + CS6 ETEC during stationary phase

**Figure 2:**
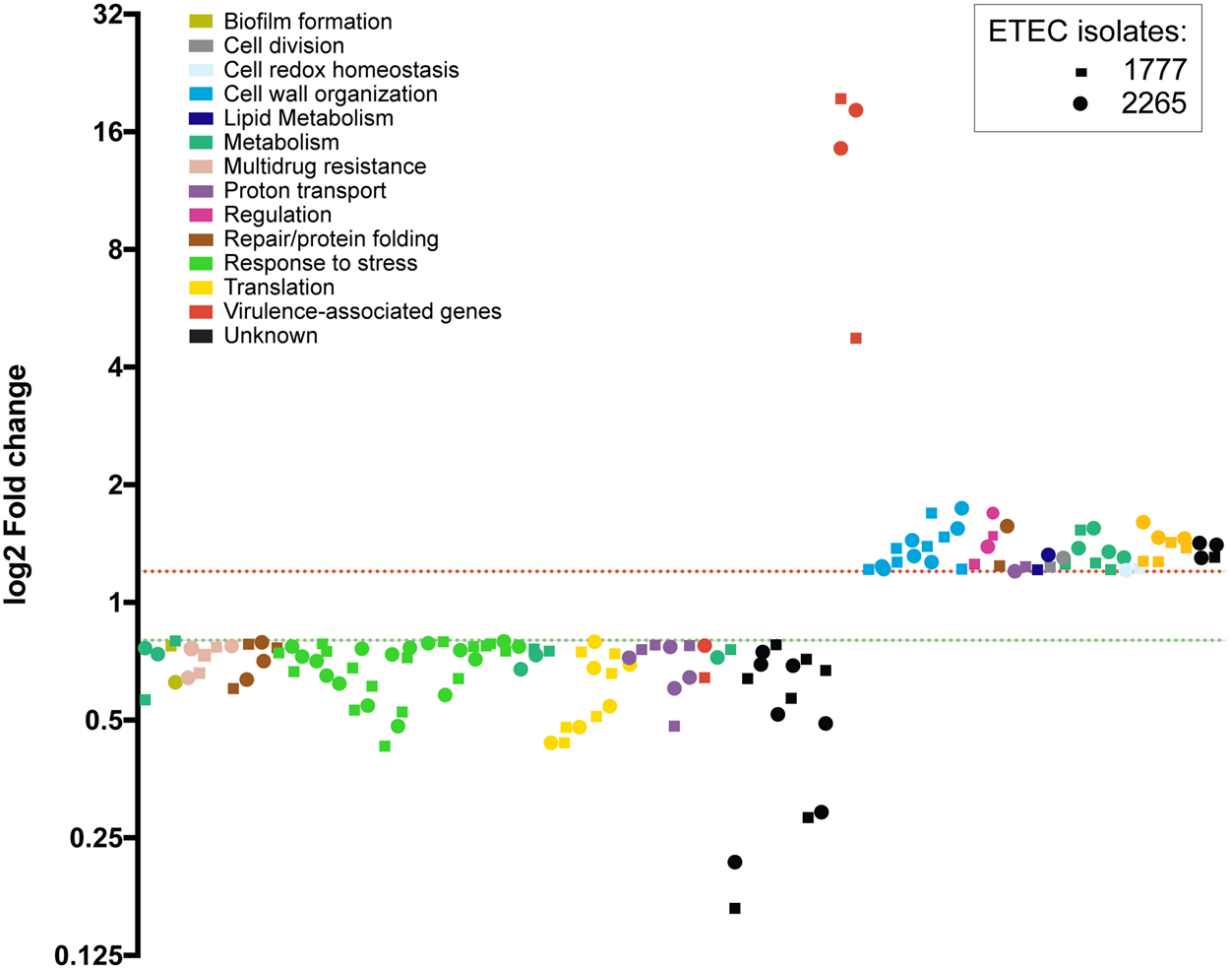
Quantitative proteomic analysis of ETEC isolates (E2265 and E1777) treated with 0.2% NaGCH. Graph shows fold change in protein abundance after NaGCH exposure; fold change <0.8 indicates a significant decrease, fold change >1.2 indicates a significant increase. Proteins were organized with GraphPad Prism 7.00 for Mac OS X based on their respective pathways.

To determine if the changes induced by NaGCH on gene expression correlated with changes in protein levels, we performed a quantitative proteomic analysis on strains E1777 and E2265 grown to stationary phase. With iTRAQ labeling and liquid chromatography combined with tandem mass spectroscopy (MM/MS), we identified 1,480 differentially produced proteins in E1777 and 1,231 differentially produced proteins in E2265 (false-discovery rate < 1%). A fold change in protein abundance of ≤0.8 (downregulated) or ≥1.2 (upregulated) (and with at least 2 unique peptides) was considered significant. Considering these criteria, NaGCH increased the abundance of 88 proteins in E1777 and decreased the abundance of 78 proteins (Supplementary Table S2). In E2265, NaGCH increased the abundance of 107 proteins and decreased the abundance 86. Overall, NaGCH increased the levels of 26 proteins common to both isolates and decreased 46 (Fig. 2, Supplementary Table S2).

### Proteomic data confirm upregulation of CS5 and CexE

**Figure 3:**
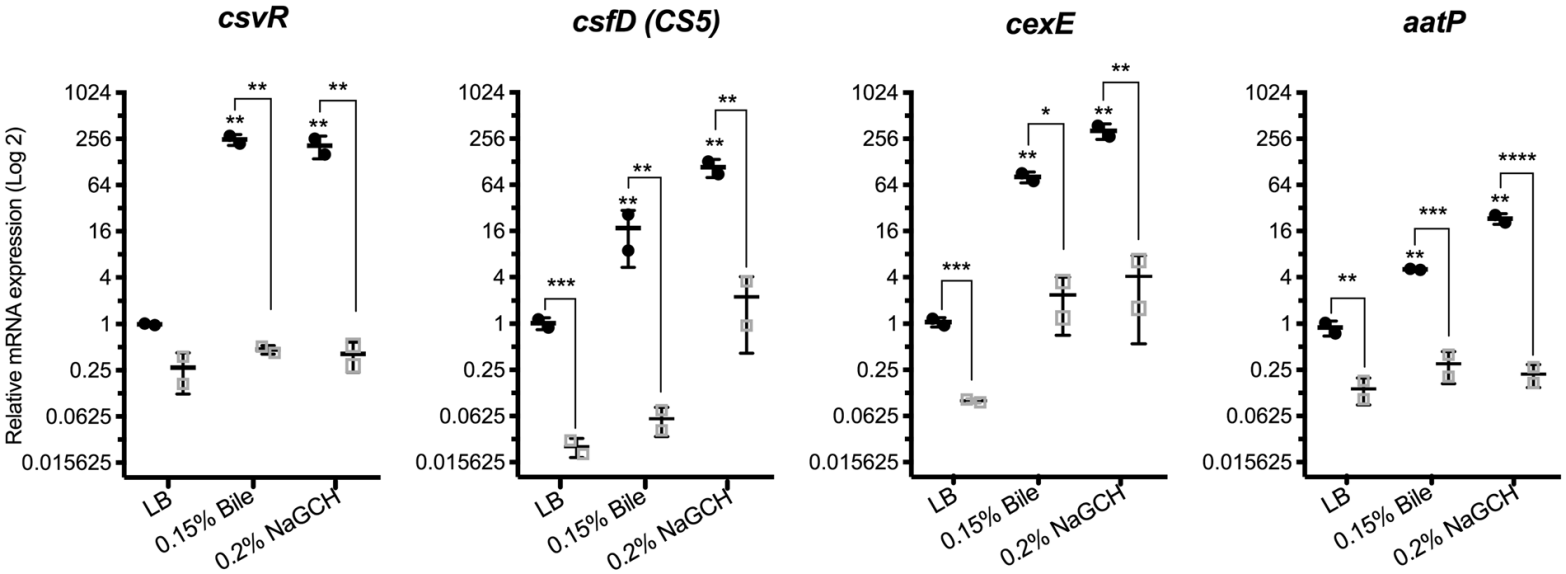
Validation of RNA-Seq and proteomic data by quantitative real-time RT-PCR. mRNA from ETEC isolates E1777 and E2265 was extracted after 3 hours or 24 hours (ON) of growth in LB, LB + 0.15% bile, or LB + 0.2% NaGCH. Relative mRNA expression values are the mean plus standard deviation (error bars) of at least three independent experiments. Asterisks indicate significant difference by one-way ANOVA (*P < 0.05; **P < 0.01; ***P < 0.001; ****P < 0.0001) using GraphPad Prism 7.00 for Mac OS X.

**Figure 4:**
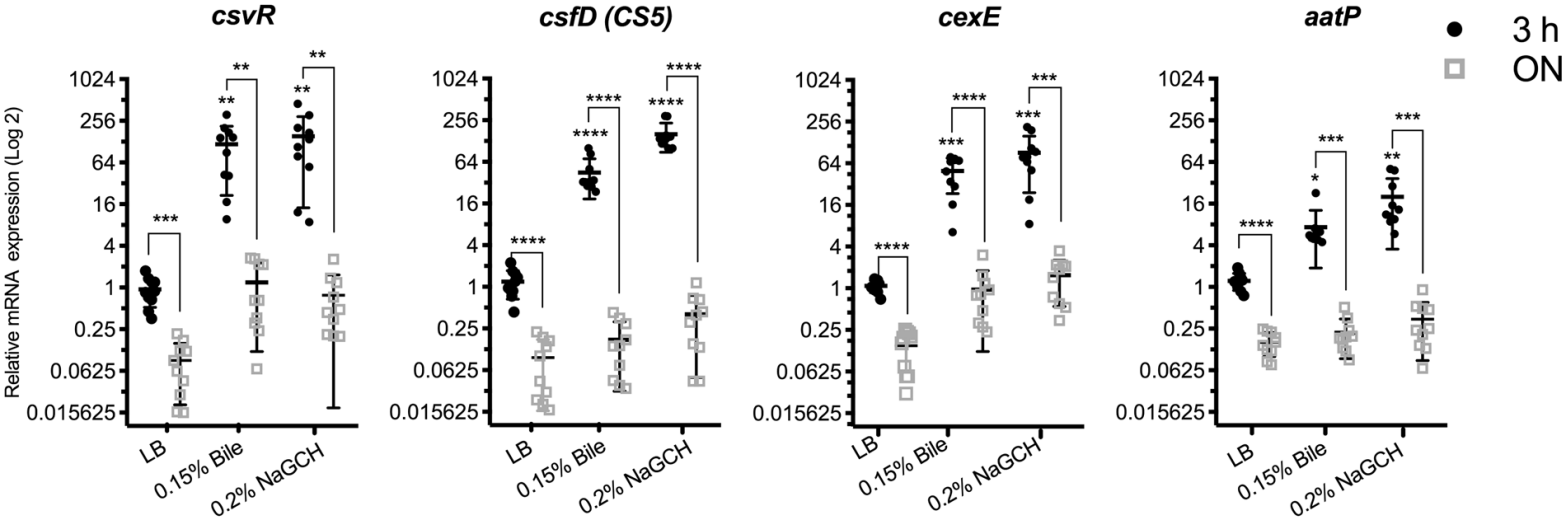
Expression of virulence-associated genes in globally distributed CS5 + CS6 ETEC strains. mRNA from 10 CS5 + CS6 ETEC isolates was extracted after 3 hours or 24 hours (ON) of growth in LB, LB + 0.15% bile, or LB + 0.2% NaGCH. Quantitative real-time RT-PCR was used to analyze differential gene expression. Relative mRNA expression values are the mean plus standard deviation (error bars) of three independent experiments. Asterisks indicate significant difference by one-way ANOVA (*P < 0.05; **P < 0.01; ***P < 0.001; ****P < 0.0001) using GraphPad Prism 7.00 for Mac OS X.

Consistent with the transcriptomic analysis, peptides of the CS5 fimbrial subunit CsfD and the outer membrane protein CexE, both virulence proteins, were most abundant after exposure to NaGCH (Fig. 2, Supplementary Table S2). However, there was no significant change in the abundance of CsvR, the proposed transcriptional activator of CS5, indicating that it was likely transiently upregulated during exponential phase. To confirm this, we measured *csvR* expression with qRT-PCR during exponential and stationary growth; we found that *csvR* was expressed during exponential growth, but expression then declined (Figs 3 and 4). We also found that NaGCH decreased the abundance of CS6, which is co-expressed in the ETEC strains in this study (Fig. 2, Supplementary Table S2). This trend was not observed in our transcriptomic analysis and could not be confirmed by qRT-PCR.

### NaGCH upregulates membrane and cell wall proteins during stationary phase

Our proteomic analysis showed that NaGCH significantly increased the abundance of several proteins involved in biofilm formation, cellulose production, and the cell envelope, including Blc and YfiO (lipoproteins anchored to the periplasmic face), outer membrane proteins BepA and YfgM, and MipA, DacA, and DacC (involved in peptidoglycan biosynthesis and structure). In addition, NaGCH upregulated the amino acid uptake/transport proteins GlnQ, GltL, PheA, and PutP (Fig. 2, Supplementary Table S2).

NaGCH also induced the production of the acetate operon repressor IclR and the global regulator Lrp (Fig. 2, Supplementary Table S2). Finally, we observed increased levels of AtpC (responsible for proton transport), FadA (fatty acid oxidation), NlpD (cell division), TrxA (redox homeostasis), and Ung (DNA repair) (Fig. 2, Supplementary Table S2).

### NaGCH represses proteins involved in stress responses, central metabolism, and translation

A number of proteins were significantly downregulated by NaGCH in both ETEC strains (Fig. 2 and Supplementary Table S2). Many these proteins are involved in different stress responses, including OsmY, YgaU, YciE, YciF, and YhbO (osmotic stress); AcnA, KatE, YdbK, YhbO, and PoxB (oxidative stress); YgiW and SodC (hydrogen peroxide stress); HdeB and GadC (pH stress); and AppA and Dps (starvation). We also observed decreases in stationary-phase 50 S (L32 and L33) and 30 S (S19, essential; S20, non-essential) ribosomal proteins, as well as the ribosome-associated proteins RMF and SRA. NaGCH treatment also decreased the production of the multidrug transporters MdtE and MdtF, which were associated to multidrug resistance, enterobactin secretion, and iron homeostasis^31^. Likewise, NaGCH decreased the abundance of the tryptophanase TnaA, which converts tryptophan into indole, ammonia, and pyruvate, allowing *E. coli* to use tryptophan as a sole source of carbon and nitrogen. Indole is a signaling molecule that represses biofilm formation and motility and regulates virulence in EHEC and EAEC^32,33^.

### The p1 ETEC plasmid contains several bile-/NaGCH-inducible virulence genes

**Figure 5:**
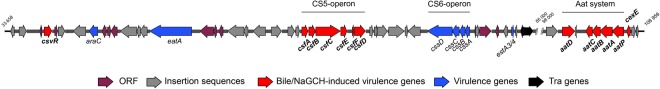
Linear representation of plasmid p1 from ETEC E2265. PacBio sequencing and analysis of the largest plasmid in E2265 (142359 bp, GenBank Accession number CP02334) with virulence-associated genes that were induced by bile and NaGCH (red arrows). Magenta arrows, ORFs; gray arrows, insertion sequences; blue arrows, virulence genes not induced by bile and NaGCH; and black arrows, Tra (transfer) genes.

We suspected that *csvR*, *csfD*, and *cexE*, the most highly induced virulence-associated genes, were plasmid borne. Therefore, we used PacBio sequencing to analyze plasmid DNA from strain E2265; we confirmed that these and other virulence-associated genes were encoded on a large 142-kb plasmid. As is illustrated in Fig. 5 *csvR* was proximally upstream of an *araC* homolog. Downstream of this gene were *eatA* (ETEC autotransporter A) and the CS5 (*csfABCEFD*) and CS6 operons (*cssABCD*), oriented head-to-head. Next to these CF operons, we identified *estA3* (heat stable toxin, STh), the *aatPABCD* operon (Aat translocator system for dispersin homolog), and *cexE* (Fig. 5). Comparing sequences, we found that E2265 *cexE* was only 72% similar to the H10407 ETEC *cexE* (acc. No. EF205439.1); however, it was 100% identical to a previously annotated sequence in the draft whole-genome sequence of another CS5 + CS6 ST ETEC strain^34^ and to a non-annotated sequence on a CS6-encoding plasmid^35^. We deposited the sequence of the E2265 plasmid at GenBank (accession no. CP023347)^36^.

### Bile and NaGCH activate virulence in CS5 + CS6 ETEC strains from a globally distributed ETEC linage

To validate our transcriptomic and proteomic data and determine if adjacent plasmid-borne virulence genes were also induced by bile/NaGCH, we performed qRT-PCR with E1777 and E2265 after 3 hours or overnight growth (ON) (Fig. 3). As expected, NaGCH significantly increased the expression of *csvR*, *csfD*, and *cexE* after 3 hours of growth. In addition, *aatP* expression was upregulated by bile and NaGCH. After overnight growth, the gene expressions of all four virulence genes were drastically reduced compared to 3 hours of growth in LB. When bacteria were exposed to bile or NaGCH, virulence gene expression showed a tendency of being higher than in the ON LB control but the expression levels significantly lower than after 3 hours of growth (Fig. 3). Hence, bile and NaGCH induction of virulence is transient and occur in exponential growth phase.

Next, we sought to address whether bile and NaGCH activate pathogen-specific genes in other CS5 + CS6 ETEC strains from the ETEC L5 lineage^37^. For these experiments, we performed qRT-PCR on 10 additional ETEC clinical isolates after 3 hours or ON growth. As with the E1777 and E2265 isolates, bile and NaGCH significantly upregulated virulence gene expression in these 10 CS5 + CS6 ETEC strains (Fig. 4). The most significantly upregulated gene was the CS5 transcription factor *csvR* (bile, 9.7- to 312.1-fold; NaGCH, 12.2- to 452.4-fold). The genes *csfD* (CS5 minor subunit) and *cexE* were also upregulated significantly by bile (*csfD*, 23.8- to 83.4-fold; *cexE*, 6.5- to 78.8-fold) and NaGCH (*csfD*, 100.6- to 293.4-fold; *cexE*, 8.5- to 214.4-fold), while *aatP* was induced to a lesser extent (bile, 4.81- to 22.7-fold; NaGCH, 8.82- to 50.7-fold). Although the ETEC strains were variably induced, our data indicate that NaGCH had a more profound effect on gene expression than bile. Similar to E2265 and E1777, after overnight growth the expression of virulence genes in LB significantly dropped, and bile or NaGCH supplementation increased their expression but no significance was found (Fig. 4). We conclude that physiological concentrations of bile and NaGCH contribute significantly to upregulating virulence genes in CS5 + CS6 ETEC during exponential growth phase.

### The bile salt NaGCH induces CS5-dependent bacterial co-aggregation

**Figure 6:**
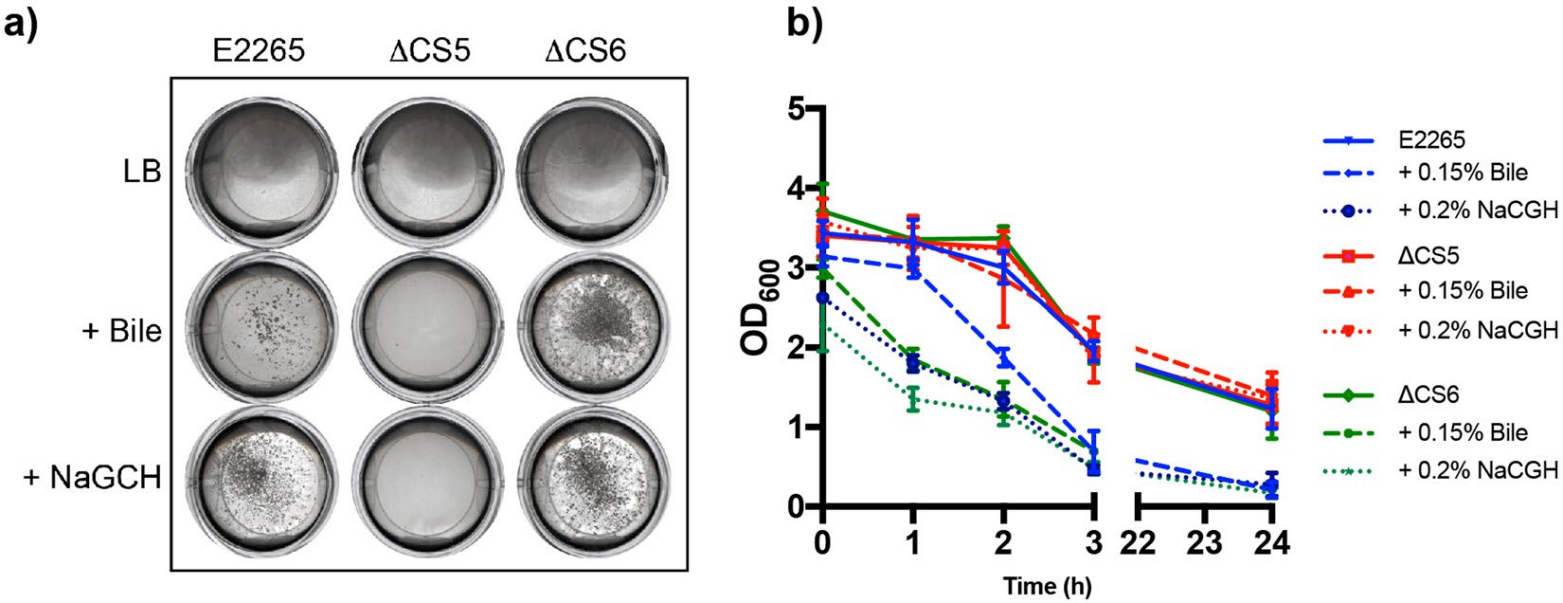
Bile and NaGCH induce bacterial co-aggregation mediated by the CF CS5. (**a**) Cell–cell aggregation and (**b**) settling patterns of ETEC E2265 and its isogenic mutants (E2265 ΔCS5 and E2265 ΔCS6) after growth in the presence of 0.15% bile or 0.2% NaGCH. Liquid suspensions of bacterial cells were left standing for 24 hours, and the OD_600_ of planktonic bacteria was measured at the times indicated and the mean and standard deviation (error bars) were plotted. Comparisons between control (E2265 in LB) with E2265 + 0.15% bile, E2265 + 0.2% NaGCH, E2265 ΔCS6 + 0.15% bile, and E2265 ΔCS6 + 0.2% showed significant differences (P < 0.001).

We observed that our bacterial cultures aggregated into clumps during growth with bile or NaGCH. This aggregation was most evident in the presence of NaGCH, where NaGCH-treated cells sedimented more rapidly than bile-treated ones (Fig. 6a and b). To determine if the CFs CS5 and CS6 played a role during bacterial co-aggregation, we generated E2265 Δ*csfABCEFD* (ΔCS5) and E2265 Δ*cssABCD* (ΔCS6) mutants and assessed them for aggregation in liquid culture in the presence of bile or NaGCH. We found that the ΔCS5 mutant did not aggregate or settle when exposed to bile or NaGCH, while the ΔCS6 did (Fig. 6a and b). Collectively, these data suggest that CS5 mediates bile- and NaGCH- dependent cell–cell aggregation.

### NaGCH induces bacterial adherence to epithelial cells

**Figure 7:**
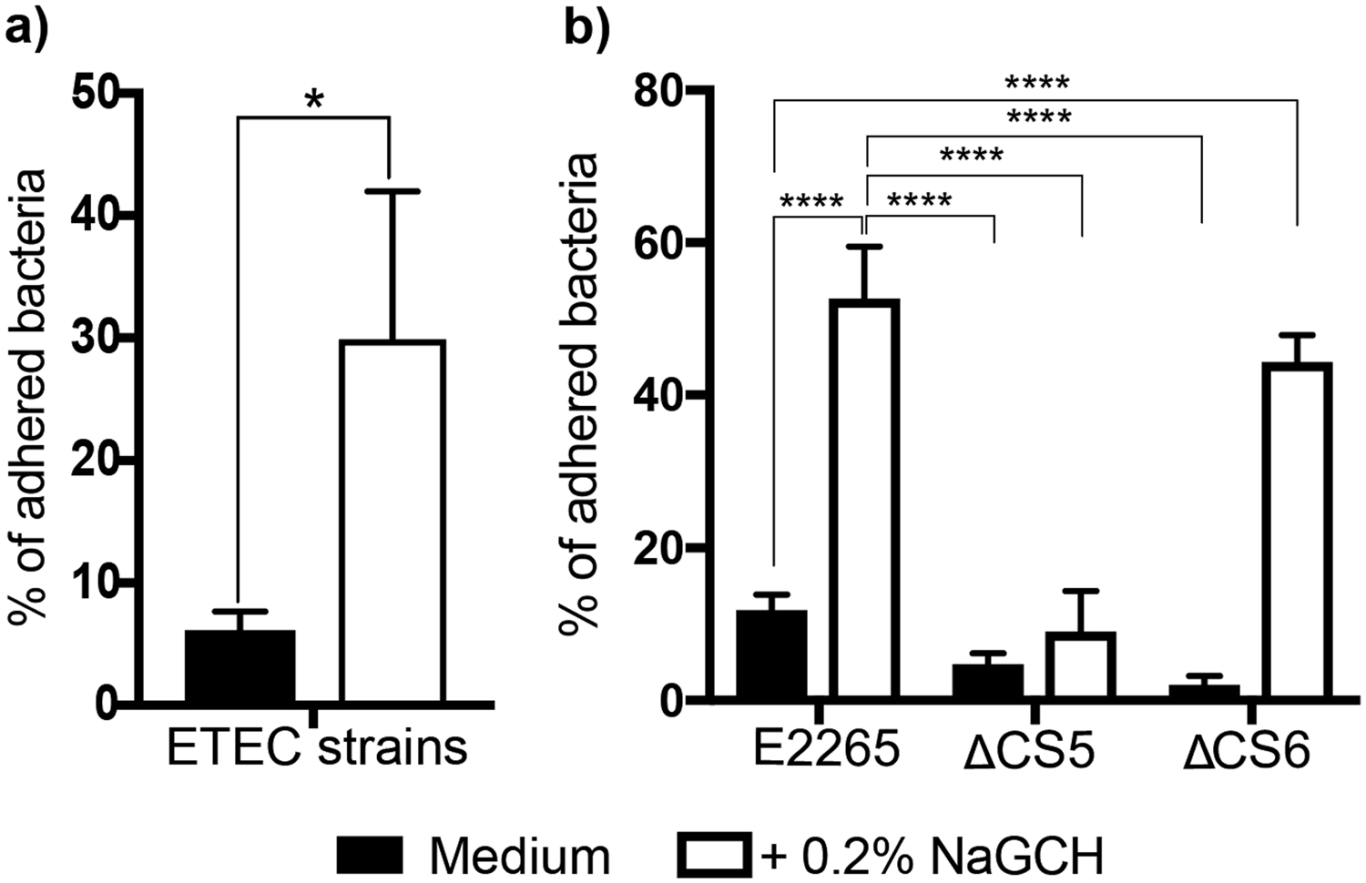
Role of NaGCH and CFs in epithelial cell adherence. Adherence assays were performed for 3 hours in DMEM with 10% of FBS (“medium”) with or without 0.2% NaGCH. Percent bacteria adhered to Caco-2 cells for (**a)** 5 CS5 + CS6 ETEC strains (E88, E1111, E1724, E1777, and E1779) and (**b)** E2265 and its isogenic mutants (E2265 ΔCS5 and E2265 ΔCS6). Assays were performed in triplicate, and graphs show the mean and standard deviation (error bars). The percentage of adhered bacteria was calculated as described in Materials and Methods. Asterisks indicate significant difference by one-way ANOVA (*P < 0.05; ****P < 0.0001) using GraphPad Prism 7.00 for Mac OS X.

Because NaGCH induced CS5 expression, we speculated that it might also affect the attachment of ETEC to intestinal epithelial cells. To evaluate bacterial attachment, we co-cultured Caco-2 cells with five CS5 + CS6 ETEC clinical isolates in the presence of 0.2% NaGCH. Indeed, we found that NaGCH significantly enhanced the ability of ETEC cells to attach to Caco-2 cells compared to untreated ETEC (Fig. 7a).

To determine if epithelial cell attachment was, like aggregation, dependent on CS5, we co-cultured Caco-2 cells with E2265 ΔCS5 or E2265 ΔCS6. We found that, compared to wild type E2265, NaGCH decreased the ability of the ΔCS5 strain to adhere to the Caco-2 cells (*p* < 0.0001) (Fig. 7b), while NaGCH did not affect attachment in the ΔCS6 mutant. These data suggest that the NaGCH-induced attachment of ETEC to intestinal epithelial cells depends on CS5.

### NaGCH does not enhance classical biofilm formation

Bile and its components are known to induce biofilm formation^38–40^. To determine if the aggregation phenotype induced by bile salts was associated with the formation of a traditional biofilm, where single cells attach to a surface, divide and develop into mature, three-dimensional structures, we assayed biofilm formation for wild type E2265, ΔCS5, and ΔCS6 cells in the presence of bile or NaGCH (Supplementary Figure S1a). Since glucose in the medium might promote biofilm formation^40^ we performed additional experiments with and without addition of 0.4% glucose. Evaluation of surface-attached bacteria by Crystal violet staining that did not show any significant biofilm induction in presence of bile salts (Supplementary Figure S1). Similarly, analysis of the red-dry-rough (rdar) phenotype and quantification of extracellular polymeric substances (EPS) using congo red (CR) and concanavalin A (ConA) staining, respectively did not show any enhanced biofilm formation (Supplementary Figure S1b and c). Addition of glucose did not influence biofilm formation. Additional gene expression analysis of four biofilm-related genes showed significant NaGCH-induced upregulation of the genes *rcdA* (regulator of biofilm master regulator CsgD) and *bsmA* (lipoprotein) while there were no significant changes in the gene expression of *csgA* (major curli subunit) and *bscB* (cellulose synthesis) (Supplementary Figure S1d). This suggested that NaGCH-induced aggregation occurred independently of the classical biofilm regulators.

### Motility is downregulated in response to bile and NaGCH

Bile and NaGCH down-regulated the expression of the flagellin gene *fliC* in E2265 and E1777. To determine if this decrease in *fliC* expression affected motility, we performed motility assays by culturing the cells on soft agar. Crude bile affected the agar such that we could not perform the assay. However, NaGCH clearly reduced bacterial motility (Supplementary Figure S2).

**Figure 8:**
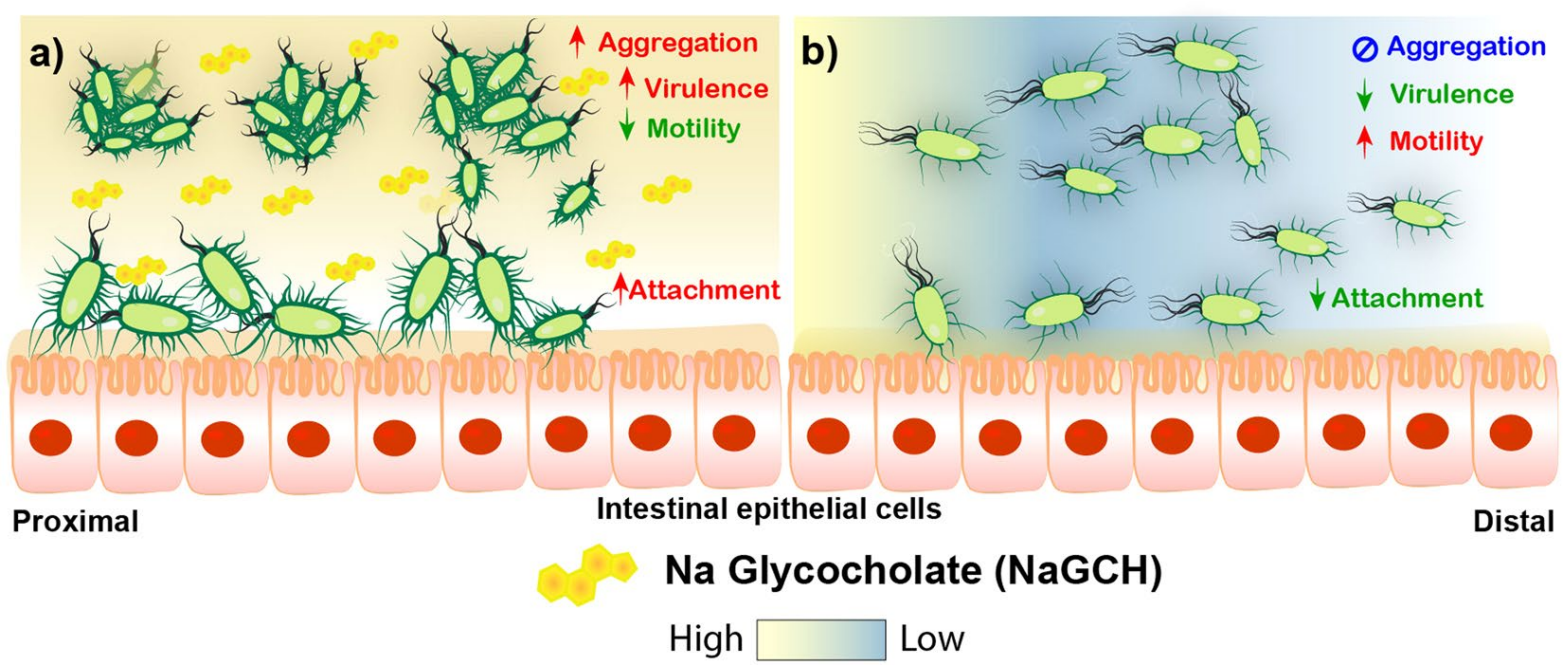
Schematic overview of NaGCH signaling during ETEC infection. (**a**) In the proximal part of the small intestine, ETEC meets a high concentration of NaGCH. This downregulates bacterial motility but activates virulence, inducing bacteria–bacteria adherence and favoring bacteria–host interactions and attachment. (**b**) We speculate that when bacteria progress through the gut and NaGCH is reabsorbed and its concentration decreases, activating bacterial motility to disperse the cells occurs.

Our results indicate that the bile salt NaGCH upregulates the expression of virulence factors in ETEC, induces bacterial aggregation, and enhances adherence to host epithelial cells (evidently a conserved feature among CS5 CS6 ETEC clinical isolates). Further, we demonstrated that the CF CS5 plays a major role in these NaGCH-dependent phenotypes (Fig. 8).

## Discussion

To successfully colonize the gut, bacterial pathogens depend on their ability to sense distinct host-derived molecules in the intestinal tract. Bile is abundant in the gut and is encountered by enteric pathogens during the early stages of infection. Besides its role in fat absorption and its antimicrobial activity, bile is well-tolerated by many microbes and can induce virulence factors in enteric pathogens^18,41–44^. Bile is a complex mix of salts, cholesterol, phospholipids, and biliverdin, but up to 40% of the dry mass of the bile used in our experiments (Oxoid) was NaGCH.

We previously reported that bile induces expression of the colonization factor CS5 in ETEC,^17,25^ and we confirmed that NaGCH was the active bile component that induced CS5 expression in a dose-dependent manner and that CS5 was expressed on the bacterial surface during acute infection^17^. In the present study, we used more powerful tools (RNA-Seq and iTRAQ-coupled LC-MS/MS) to demonstrate that bile and NaGCH modulate the global transcriptome and proteome of ETEC. Interestingly, NaGCH strongly induced the expression and translation of several plasmid-encoded genes located close to each other on a large virulence plasmid. We also found that CS5 mediates bacterial–bacterial and bacterial–host cell interactions in response to NaGCH.

Bile secreted into the intestinal lumen acts a barrier to microbes, preventing them from colonizing the intestines. Bile salts are highly effective detergents and antimicrobial agents; they solubilize lipids during digestion and exert a major selective pressure that modulates the gut microbiome^45^. Commensal and pathogenic enteric bacteria have evolved mechanisms to protect them from the antimicrobial effects of high concentrations of bile salts in the proximal small intestine^23^. *Salmonella enterica* serovar Typhimurium^40,46^, *V. cholerae*^47^, and enterohemorrhagic *E. coli* (EHEC)^48^ efficiently pump bile salts and other toxic compounds out of the cell via efflux transporters *e.g* AcrAB-TolC,. In this study, where we used 0.15% bile and 0.2% NaGCH, the genes and corresponding proteins encoding this efflux system showed a tendency of induction but were not significantly up-regulated using our criteria, nevertheless our results were consistent with another study that demonstrated a two-fold upregulation in response to bile^49^. Further, we previously described the upregulation of *tolC* transcription in ETEC expressing CS6 but not in ETEC expressing CS5 + CS6^50^. This is consistent with our current results and suggests that *tolC* induction might differ between ETEC lineages.

We did not observe growth inhibition in the presence of the concentrations used (data not shown). Other studies have also found that physiological concentrations in the small intestine do not affect growth^40^. The proteome data revealed that exposure to bile and NaGCH still had effect on expression of several proteins involved in cell wall composition and membrane stability after 24 hours of exposure. This might be part of the ETEC bile stress adaptation and shows that even physiological concentrations of bile and NaGCH induce big changes in the proteome of ETEC.

Many bacterial pathogens are capable to form biofilms in response to bile salts and several gram-negative pathogens *i.e. V. cholerae*^51^, *Shigella flexneri*^40^, and *Salmonella spp*^38,52,53^ respond with enhanced biofilm formation in presence of bile. In CS5 + CS6 ETEC, the RNA-seq data showed that *bsmA* and *yodD* were the only biofilm-associated genes transcriptionally induced by NaGCH. In addition, reduced protein abundance of the biofilm regulator McbR, involved in *E. coli* biofilm development^54^ and increased expression of the *yoaD* gene involved in the degradation of cyclic di-GMP, a signal molecule that triggers cellulose biosynthesis found in our analysis correlates with the lack of significant induction of the phenotypic formation of a traditional biofilm in response to bile and NaGCH. This model involves the attachment of single planktonic bacteria to a surface and further development to a mature biofilm^55^. Nevertheless, we demonstrated that NaGCH induced formation of multicellular aggregates and increased bacterial adherence to epithelial cells and describe that this phenotype occurs in ETEC. Indeed, aggregates has been proposed to be an alternative model of biofilm formation in *Pseudomonas aeruginosa*^56^, where instead of single planktonic cell, multicellular clumps land on surfaces and initiate the biofilm formation process during its early development. In a study of *S*. *flexneri*^40^, the authors reported that bile salts induced bacterial aggregation, while both glucose and bile were needed for EPS and biofilm formation^40^. Contrary to these results we found that addition of glucose did not promote biofilm formation or EPS in CS5 CS6 expressing ETEC. In light of several recent reports of aggregation phenotypes in bacteria further studies are clearly needed to clarify the impact of aggregation during adherence to the host cells and biofilm formation as well as pathogen specific differences in responses to host cues. Similar to other pathogenic *E. coli* where aggregation is mediated by adhesins such as adherence fimbriae AAF/I and AFF/II in EAEC, or bundle-forming pili in EPEC, we could show that the colonization factor CS5 not only plays an essential role during bacterial adherence to epithelial cells, but also is responsible of the aggregative phenotype of ETEC during bile exposure. Hence there seems to be a dual function of both adherence and aggregation linked to fimbriae expressed by pathogenic enteric bacteria, which should be further explored in relation to virulence.

Biofilm and/or adhesion to epithelium is often linked to increased motility. However, we observed reduced motility in soft agar and downregulation of the *fliC* gene in response to NaGCH. These results are corroborated by studies of EHEC, EPEC and Salmonella^57–59^, while *Vibrio choleare* and *Campylobacter* respond to bile with increased motility^58^. The results indicate that enteric pathogens respond to bile using different defense mechanisms. *E.g V. cholerae* induce motility to enable the bacteria to swim closer to the epithelium. At the epithelium where the bile concentrations are reduced *V. cholerae* instead induce toxin and adhesion genes and repress motility. The results from pathogenic *E. coli* instead suggest that bacteria form aggregates as a defense mechanism against bile stress. Since the aggregating factors also often mediate attachment to the host epithelium this mechanism might allow pathogens to adhere to the epithelium in large constellations and thus develop to a biofilm, which might facilitate infection.

Enteropathogenic bacteria hence might use bile as a signaling molecule to modulate virulence. The most intriguing result of our study was the substantial increase in the expression and production of CS5 and CexE, both of which were upregulated more than 100-fold by NaGCH. Likewise, transcription of the plasmid-encoded AraC-like transcriptional regulator *csvR* was upregulated and this activation was remarkably high during bacterial exponential phase. During stationary phase, bacteria encounters nutrient limitations, accumulation of toxic by-products and transcriptional adaptation due to change in σ-factors to cope with stress and starvation^60,61^. These, together with the stress of bile could potentially have disfavored virulence activation. Future investigations are needed to determine if these factors are transiently induced using appropriate controls. We showed previously that bile induces different strain-specific responses in the transcription and secretion of the heat stable ETEC toxin ST and its variants^50^. Specifically, the STa5-ST variant present in CS6-ETEC strains was induced by bile, while *estA3/4* encoding the ST variant ST3/4 expressed by in CS5 CS6 ETEC was not^50^. The present study confirmed these results and bile and NaGCH did not up-regulate toxins genes *estA3/4* or *eltAB* encoding LT.

However, the effect of bile on bacterial virulence varies. For instance, bile induced the expression of the afimbrial LDA adhesion in atypical EPEC^39^, while bile downregulated *stx2* and genes in the locus of enterocyte effacement in *E. coli* O157:H7^57^. Similarly, *V*. *cholerae* exhibits a complex response to bile. In early studies of the *V. cholerae* O1 classical biotype strain O395, bile decreased cholera toxin (CT) production^62^. When the bacteria were exposed to cholate only, CT production was induced directly by ToxR, and CT repression was induced by the unsaturated fatty acids in bile^63^. Exposure to specific bile components such as taurocholate and glycocholate also promote virulence in the *V. cholerae* El Tor biotype^18^; whereas, deoxycholate and chenodeoxycholate do not. This might explain why CS5, *cexE*, and *csvR* were upregulated almost two-fold in the presence of pure NaGCH compared to crude bile, which consists of a mix of bile salts, unsaturated fatty acids, and other components. These studies suggest that bacterial pathogens recognize a specific set of bile salts and interpret them as signals to either activate or reduce virulence^64^.

RNA-Seq of a CS1 + CS3 ETEC reference strain showed that the plasmid-encoded AraC-like transcriptional regulator *peaR* was one of the most differentially expressed genes in the presence of bile salts^34^. This is in line with our finding concerning the *araC*-like transcription factor *csvR* and suggests that a general bile response may be mediated by the activation of these similar transcription factors. By using PacBio analyses of the E2265 plasmid, we found that another AraC-family transcriptional regulator, the ETEC autotransporter encoded by *eatA*, the ST gene (*estA*3/4), and an *aat* translocator system were situated downstream of *csvR*. Interestingly, Santiago, *et al*.^65^ described a similar genetic organization with similar conserved features, although the precise location and genes varied among EAEC, ETEC H10407, (CFA/I) and ETEC 1392/75, (CS1, and CS3), Shiga-toxin producing EAEC, and *Citrobacter rodentium*. Altogether, we suggest that pathogens may harbor a conserved set of genes that respond to bile. Our further analysis confirmed that NaGCH induced the Aat translocator operon on the ETEC plasmid, while the nearby genes *eatA* and *araC* were not induced. The ETEC Aat translocator is homologous to the Aat (enteroaggregative ABC transporter) complex involved in the translocation of dispersin (Aap, anti-aggregation protein) in EAEC, and it is controlled by the major AraC-like transcriptional activator AggR^51^. AggR is similar to CsvR in ETEC. Because Aat_ETEC_ genes are upstream of *cexE*, we hypothesize that this translocator is involved in CexE transport, considering that CexE contains a secretory signal peptide that is removed during translocation across the cytoplasmic membrane through the general secretory pathway. However, this should be analyzed further. Although the function of *cexE* is unknown, it is regulated by CfaD/Rns, which regulates the CS1 pilus in ETEC strains expressing the colonization factors CFA/I or CS1 + CS3; it is also only present among ETEC strains. Consequently, a previous study suggested that it was a virulence gene^30^ but not further studies on its role in virulence were performed. Because *cexE* expression increased significantly in the presence of bile and NaGCH, we suggest a role for *cexE* during intestinal infections.

In summary, prior to adhesion and infection, bacterial pathogens inevitably come into contact with various concentrations of bile salts in the gut lumen; the concentrations are high at the proximal region of the small intestine and much lower in the distal region of the small intestine and into the large intestine (Fig. 8). Our data indicate that LT STh CS5 + CS6 ETEC interprets the levels of NaGCH in the proximal small intestine as a signal to induce gene expression aiming to colonize this specific site.

## Materials and Methods

### Bacterial strains

The clinical ETEC isolates E1777 and E2265, which both express the CS5 and CS6 colonization factors and the LT and STh enterotoxins, were used for transcriptomic and proteomic analyses (Supplementary Table S3). The strains were isolated from stool samples from adults with acute diarrheal disease at the hospital of the International Centre for Diarrheal Disease Research (icddr,b) in Dhaka, Bangladesh, in April 2005 and March 2006, respectively^17,66^. The toxin profile was determined with ELISA and PCR, and the colonization factor profile was determined with dot-blot assays for various CFs^25^. Both strains have been sequenced and belong to the global phylogenetic lineage L5 and are multilocus sequence type 443^37,66^. Clinical isolates from the University of Gothenburg ETEC collection were used for additional studies of gene expression and cell adhesion (Supplementary Table S3). All strains were kept at −70 °C in glycerol stocks and streaked onto blood agar plates to confirm purity before experimentation.

### RNA sample preparation

Bacteria were grown from frozen stocks overnight on blood agar plates at 37 °C. Starting cultures were prepared by growing 10 colonies in 10 ml of LB medium with shaking to an OD_600_ of 0.8 (*i.e*. 10^9^ bacteria/ml). The cultures were diluted 1:100 into a final volume of either 20 ml LB medium, LB supplemented with 0.15% (w/v) ox-bile extract (Oxoid), or LB supplemented with 0.2% (w/v) sodium glycocholate hydrate (NaGCH) (Sigma-Aldrich) and grown in 250 ml Erlenmeyer flasks under aerated conditions at 37 °C (200 rpm). The cultures were followed for 5 hours to determine the growth kinetics. Samples were collected for RNA extraction after 3 hours of growth, when cultures had reached mid-logarithmic growth and an OD_600_ of 2.5 to 3. The samples, containing 1 × 10^9^ bacteria, were immediately mixed with a 2X volume of RNAProtect^®^ (Qiagen), incubated at room temperature for 5 minutes, and centrifuged for 10 minutes at 13,000 rpm. Bacterial pellets were stored at −70 °C until RNA extraction.

To prepare total RNA, bacterial cells were lysed with lysozyme (1 mg/ml) and treated with proteinase K using the RNeasy Mini Kit (Qiagen), according to the manufacturer’s instructions. RNA purity was confirmed with agarose gel electrophoresis, and the concentration was determined with a NanoDrop ND-1000 (NanoDrop Technologies). Aliquots of RNA were reserved for cDNA preparation and qRT-PCR. For RNA-Seq, RNA was extracted in parallel in duplicate or triplicate from the remaining samples for each culture condition; volumes corresponding to 20 µg RNA were pooled and precipitated overnight at −20 °C with a 1:10 volume of 3 M NaAc and a 2X volume of 99.5% ethanol. The RNA was centrifuged for 30 minutes at 13,000 rpm, and the pellet was washed with 70% ethanol and centrifuged again. The pellets were covered with 200 µl 99.5% ethanol and stored at −70 °C until shipment to Shenzhen, China. The RNA integrity, quality, and concentration were validated upon arrival to the sequencing facility (Beijing Genome Institute, Shenzhen, China) using an Agilent 2100 Bioanalyzer (Agilent Technologies). The RIN values were above 9.8 for all samples.

### Quantitative real-time reverse-transcription PCR (qRT-PCR)

For each sample, cDNA was prepared from 600 ng RNA using the QuantiTect Reverse Transcription Kit (Qiagen). The final volumes of the cDNA samples and RT(−) controls were 20 μl, and both were stored at −20 °C until use. QRT-PCR was performed with primers specific for *csvR, csfD, cssD, cexE, aatP*, and *gapA* (Supplementary Table S3). The program for qRT-PCR was run on 20-μl reactions using standard conditions for the LightCycler® 480 Instrument II (Roche Molecular Diagnosis), with 30 ng cDNA, 8 pmol of each primer, and 10 μl Power SYBR® Green PCR Master Mix (Applied Biosystems). Transcript levels were normalized to the house-keeping gene *gapA* using the 2−ΔΔCt method; qRT-PCR data for each experimental group are expressed as the change in expression relative to bacteria grown in LB. For ON experiments, transcript levels were expressed relative to the gene expression of bacteria grown in LB at 3 hours. All values reported are the mean of at least three independent experiments.

### RNA-Seq

For RNA-Seq, rRNA was depleted with the Illumina Ribo-Zero rRNA Removal Kit for bacteria, and Illumina libraries were generated using the TruSeq protocol as described by the manufacturer. The libraries were sequenced with the Illumina Hiseq. 2000 system with single-end 100-bp read lengths; approximately 34 million reads were generated for each sample. Sequences from the raw FASTQ files were first trimmed to obtain high-quality reads, using a Phred score cut-off of 30. Reads were then assembled with SOAPdenovo software (http://soap.genomics.org.cn/soapdenovo.html). CAP3 was used to assemble the unigenes from different samples to form a single set of non-redundant unigenes. Unigene sequences with hits in the first or second databases were not searched in subsequent databases. Then, BLAST results were used to extract coding regions (CDS) from Unigene sequences and translate them into peptide sequences. The BLAST results were then used to train ESTScan^67^. CDS of unigenes with no hits in the BLAST search were predicted with ESTScan and then translated into peptide sequences.

Unigenes were functionally annotated for protein sequence similarity and via KEGG pathway, COG, and gene ontology (GO) analyses. All unigene sequences were searched in protein databases (Nr Swiss-Prot, KEGG, and COG) using BLASTX (e-value < 0.00001). The Blast2GO program was used to obtain GO annotations of the unigenes, and WEGO software^68^ was used to functionally classify all unigenes.

Differential gene expression for the experimental conditions (LB vs. LB + Bile and LB vs. LB + NaGCH) was calculated using the fold-change values per gene and per strain (E1777 and E2265) obtained from the RPKM values and later log2-transformed. A 3-fold change in gene expression (log2 ≤ −1.58 and log2 ≥ 1.58) was considered statistically significant.

The R tool gplots::heatmap.2 was used to plot the heatmap of significantly differentially regulated genes. Genes were organized by signaling pathway, and each pathway was assigned a color (Fig. 1).

### Growth conditions, protein sample preparation, and isobaric labeling for quantitative mass spectrometry (iTRAQ-coupled LC-MS/MS)

Proteomic analyses were performed on duplicate replicate samples; isobaric tags were used for relative and absolute quantitation (iTRAQ) coupled with nano-liquid chromatography (LC) and analysis by tandem mass spectrometry (MS/MS).

Starter cultures were prepared by growing 3 colonies in 10 ml LB medium for 3 hours at 37 °C with shaking. Cells from each respective starter culture (E1777 and E2265) were inoculated into two sets of three 250 mL flasks containing 25 ml LB medium with or without 0.2% (w/v) NaGCH (Sigma-Aldrich) for a final concentration of 10^7^ bacteria/ml (based on the OD_600_ value). The cultures were grown to stationary phase at 37 °C, and duplicate cultures were pooled for a total sample volume of 5 to 6 ml. The resulting 8 samples (2 × E1777-LB, 2 × E1777-NaGCH, 2 × E2265-LB, 2 × E2265-NaGCH) were centrifuged at 10,000 rpm for 5 minutes, and the pellets were washed carefully 3 times in PBS and stored at −20 °C.

### Proteomic analysis with iTRAQ

Proteomic analyses were performed at the Proteomic Core Facility at the Sahlgrenska Academy, Gothenburg University. Bacteria samples were homogenized in 300 µl TEAB (8 M urea, 50 mM triethylammonium bicarbonate) using the FastPrep®−24 instrument (MP Biomedicals). An equal volume of lysis buffer (50 mM TEAB, 8 M urea, 4% CHAPS, 0.2% SDS, 5 mM EDTA) was added, and the total protein concentration was determined with the Pierce™ 660 Protein Assay (Thermo Fisher Scientific). Aliquots containing 100 μg protein were reduced and alkylated according to the manufacturer’s instructions (AB Sciex, iTRAQ Reagents Multi [4]-Plex Kit). Samples were digested with trypsin (1:25, trypsin:protein; Promega) overnight in solution at 37 °C. The resulting peptide samples were labeled with the iTRAQ 4-plex reagents and pooled into two independent sets. The 2 sets were fractionated into 18 fractions by strong cation exchange chromatography (ÄKTA-system, Amersham-Pharmacia) on a PolySULFOETHYL A™ column (100 × 2.1 mm, 5 µm, 300 Å; PolyLC Inc.) over 40 minutes (0% to 100% 500 mM ammonium formate, pH 2.8 in 20% ACN).

Each fraction was desalted using PepClean C18 spin columns (Thermo Fisher Scientific) according to the manufacturer’s instructions. Samples were analyzed with an LTQ-Orbitrap Velos mass spectrometer interfaced to an Easy-nLC system (Thermo Fisher Scientific). Peptides were separated on a C18 analytical column (210 × 0.075 mm ID, 3 μm Reprosil-Pur C18-AQ particles, Dr. Maisch, Germany) over a 70-min gradient from 5% to 80% ACN in 0.2% formic acid. The MS scans were performed at a resolution of 30,000 with a mass range of *m/z* 400–1800. MS/MS analysis was performed in a data-dependent mode with a resolution of 7,500 and *m/z* 120–2,000, with the top 10 most abundant doubly or multiply charged precursor ions in each MS scan selected for MS/MS fragmentation. An inclusion list containing eight theoretical tryptic peptides from the protein of interest were used during the analysis. Dynamic exclusion was set to 30 seconds.

For relative quantification, the raw MS data files for each iTRAQ set were merged with Proteome Discoverer software v. 1.3 (Thermo Fisher Scientific). The database search was performed with the Mascot search engine (Matrix Science) and included the *Escherichia coli* Swiss-Prot Database (updated October 2011; Swiss Institute of Bioinformatics, Switzerland) and an in-house database containing the proteins of interest (e.g., variant of *cexE* in E2265). The search used an MS peptide tolerance of 5 ppm and an MS/MS tolerance for identification of 0.5 Da. Tryptic peptides were accepted with 1 missed cleavage and variable modifications of methionine oxidation, cysteine methylthiol and fixed modifications of N-terminal iTRAQ4plex and lysine iTRAQ4plex were selected. The detected-peptide threshold in the software was set to a 1% false-discovery rate by searching against a reversed database, and identified proteins that shared sequence were grouped to minimize redundancy. The quantification was normalized using the protein median, and missing values were replaced with minimum intensity. Only peptides unique for a given protein were considered for relative quantitation, excluding those common to other isoforms or proteins of the same family. The results were then exported into Excel for manual data interpretation.

Proteins were considered to be significantly upregulated or downregulated when the fold change between LB and LB + NaGCH was ≥1.2 and ≤0.8 with at least two unique peptides.

### Deletion of CS5 and CS6 operons

The CS5 and CS6 operon deletion mutants were constructed in strain E2265 using the λ-Red recombinase system^69^. Plasmid pKD4 was used as a template to amplify the kanamycin (kan) resistance cassette with primers cs5mutF/cs5mutR or cs6mutF/cs6mutR, incorporating 60 bp of homology to the 5′ and 3′ ends of the CS5 or CS6 operons. The purified PCR product was electroporated into E2265 harboring pKD46. The mutants were selected on LB plates with kanamycin at 37 °C and confirmed by PCR and DNA sequencing. Plasmid pKD4 was used as a template to amplify the kanamycin (kan) resistance cassette with primers cs5mutF/cs5mutR or cs6mutF/cs6mutR, incorporating 60 bp of homology to the 5′ and 3′ ends of the CS5 or CS6 operons. The purified PCR product was electroporated into E2265 harboring pKD46. The mutants were selected on LB plates with kanamycin at 37 °C and confirmed by PCR and DNA sequencing.

### Cell culture assays

Caco-2 cells (human colon adenocarcinoma cells) were obtained from the American Type Culture Collection and used in their terminally differentiated state to mimic mature small intestine enterocytes. Caco-2 cells were grown and maintained in Dulbecco’s modified Eagle’s medium (DMEM, Sigma-Aldrich) supplemented with 10% (v/v) fetal bovine serum (FBS) at 37 °C in an atmosphere of 5% CO_2_/95% air with constant humidity. For the experiments, Caco-2 cells were seeded into 24-well tissue culture plates at 25,000 cells/cm^2^ and grown over 15 days to obtain a monolayer of differentiated and polarized cells; the culture medium was changed every 3 days. Monolayers were infected with bacteria by replacing the medium with 500 μl of a suspension of DMEM + 10% FBS with 1 × 10^7^ CFU/ml bacteria (5 × 10^7^ CFU/well) with or without 0.2% NaGCH. After 3 hours of infection at 37 °C in 5% CO_2_, the cells were washed 3 times with PBS and lysed with Triton-X 0.1% for 10 minutes at 37 °C. The lysates were collected, and different dilutions were plated on LB agar and incubated for 24 hours at 37 °C to determine the CFU. Each experiment was carried out in duplicate and repeated three times.

### Crystal violet biofilm assay

Biofilm assays were performed on microtiter plates as described previously^70^, with some modifications. Overnight cultures were inoculated 1:100 into fresh LB medium, LB + 0.15% bile salts, or LB + 0.2% NaGCH. 100 μl of each diluted bacterial cultures were grown in a round-bottom 96-well polystyrene microtiter plate (Corning® Cat. No. CLS3795 Sigma). Planktonic growth was determined by measuring the absorbance (600 nm); wells were then rinsed with water, and attached cells were stained with 125 μl crystal violet (Sigma-Aldrich). Stained cells were solubilized with 200 μl 80% ethanol, and 125 μl of the solution was transferred to a flat-bottom 96-well polystyrene microtiter plate (Corning®, Cat. No. CLS3595 Sigma). Absorbance (540 nm) was measured with a SpectraMax i3x spectrophotometer (Molecular Devices). For each experiment, the absorbance of the blank control with crystal violet was subtracted from each experimental sample. Six replicates were used in each experiment, and six independent experiments were performed.

### Semi-quantitative detection of extracellular polymeric substances (EPS)

EPS matrix detection was performed as described previously 72. In brief, 130 l diluted bacterial cultures (1:50 from an overnight bacterial) in LB, LB + 0.15% bile or LB + 0.2% NaGCH were grown in a black 96-well clear flat bottom polystyrene plate (Corning® Cat. No. CLS3603 Sigma) overnight at 37 °C. Planktonic bacteria was transferred to a new 96-well polystyrene microtiter plate (Corning®, Cat. No. CLS3595 Sigma). Wells in the black plate were fixated with formaldehyde/glutaraldehyde, rinsed with PBS and stained with 150 l of Concanavalin A (ConA) conjugated to fluorescein isothiocyanate (FITC) (25 g/ml) for 15 min. After rinsing with PBS, the amount of retained ConA-FITC was measured using a SpectraMax i3x spectrophotometer (Molecular Devices) at 480 nm. This assay was performed in four independent experiments.

#### Phenotypic testing for the rdar morphotype

Bacterial strains were grown on LB-no salt agar (LBns) overnight at 37 °C and re-suspended in PBS. The bacterial suspension was adjusted to an OD_600nm_ of 3 and 5 μl was spot inoculated onto LBns agar supplemented with Congo red (40 μg/ml) and Coomassie brilliant blue (20μg/ml). The plates were additionally supplemented with either 0.15% bile or 0.2% NaGCH. Colony morphology was observed after incubation at 37 °C for 48 hours; photographs were taken after incubation.

### Motility assay

Swimming media consisted of 1% Tryptone, 0.5% NaCl and 0.25% agar with and without 0.2% NaGCH were prepared. 10 ml overnight cultures of E1777 and E2265 were concentrated 2-fold by centrifugation, washed and resuspended in PBS. 2 μl of the bacterial cell suspension was inoculated by inserting the tip of the pipette at the center of the plate, approximately 2–3 mm inside the swimming agar. Images were obtained after 8 hours of incubation at 37 °C. Three independent experiments were performed.

## Electronic supplementary material

Supplementary Information

Supplementary Tables
