# Supplementary material for: The bile salt glycocholate induces global changes in gene and protein expression and activates virulence in enterotoxigenic Escherichia coli
Source: Sci Rep. 2019 Jan 14;9:108. doi: 10.1038/s41598-018-36414-z (PMC6331568; doi:10.1038/s41598-018-36414-z)
Supplement: Supplementary file 1 — Supplementary Information [file 41598_2018_36414_MOESM1_ESM.pdf]

Supplementary Information

The bile salt glycocholate induces global changes in gene and protein expression and activates virulence in enterotoxigenic *Escherichia coli*

Authors and affiliations: Enrique Joffre [a*], Matilda Nicklasson [b], Sandra Álvarez-Carretero [a,] Xue Xiao [c], Lei Sun [a], Intawat Nookaew [d], Baoli Zhu [c e f], and Åsa Sjöling [a]

[a] Department of Microbiology, Tumour and Cell biology, Karolinska Institutet, Stockholm, Box 280, S-17177, Stockholm, Sweden

[b] Institute of Biomedicine, Department of Microbiology and Immunology, University of Gothenburg, Box 435, S-405 30 Göteborg, Sweden

[c] CAS Key Laboratory of Pathogenic Microbiology & Immunology, Institute of Microbiology, Chinese Academy of Sciences, Beijing, China

[d] Department of Biomedical Informatics, College of Medicine, The University of Arkansas for Medical Sciences, Little Rock, AR 72205, USA

[e] Collaborative Innovation Center for Diagnosis and Treatment of Infectious Diseases, The First Affiliated Hospital, College of Medicine, Zhejiang University, Hangzhou, China

[f] Department of Pathogenic Biology, School of Basic Medical Sciences, Southwest Medical University, Zhongshan Road, Luzhou, Sichuan, China

* Corresponding author:

Enrique Joffre (enrique.joffre@ki.se)

FIGURES

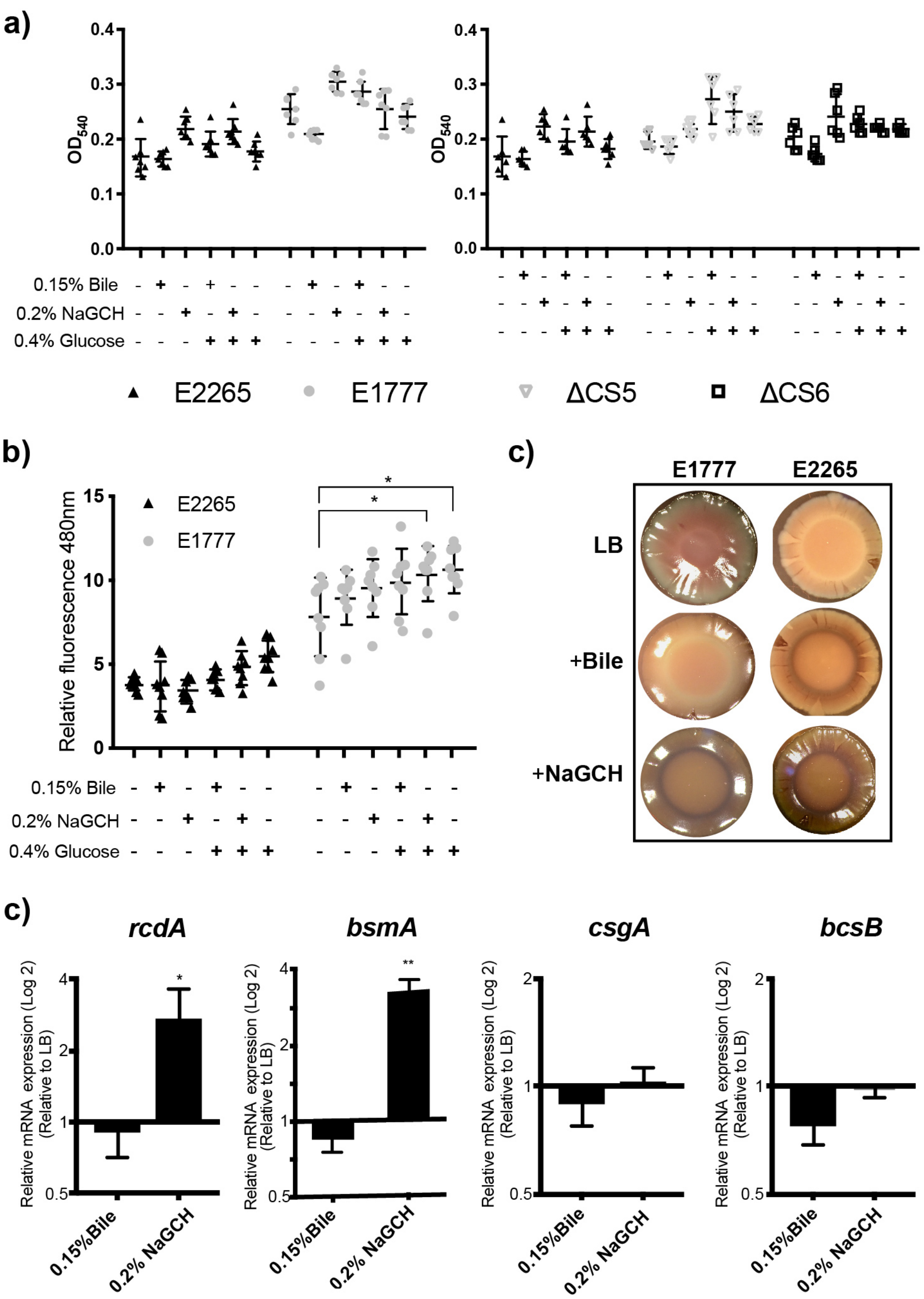

Figure S1. Influence of bile salts on biofilm formation. a) Quantification of biofilm formation in E1777, E2265, E2265 ΔCS5, and E2265 ΔCS6. Surface-adhered biofilms were quantified by measuring the OD_{540} of ethanol-solubilized crystal violet (CV) after biofilm staining. **b)** Quantification of EPS by FITC-ConA staining in E1777 and E2265. Fluorescence was measured at 488 nm. At least four independent experiments of CV and FITC-ConA assays were performed and average values are

plotted plus standard deviation (error bars). **c)** Rdar morphotype formation of E1777 and E2265 in presence of bile or NaGCH. **d)** Expression of biofilm-associated genes. mRNA was extracted after 3 hours of growth in LB, LB + 0.15% bile, or LB + 0.2% NaGCH. Subsequently, real-time quantitative PCR was performed to analyze differential gene expression. Relative mRNA expression values are the mean plus standard deviation (error bars) of at least three independent experiments. *P*-values, (*, $P < 0.05$; **, $P < 0.01$) were determined by one-way ANOVA using GraphPad Prism 7.00 for Mac OS X.

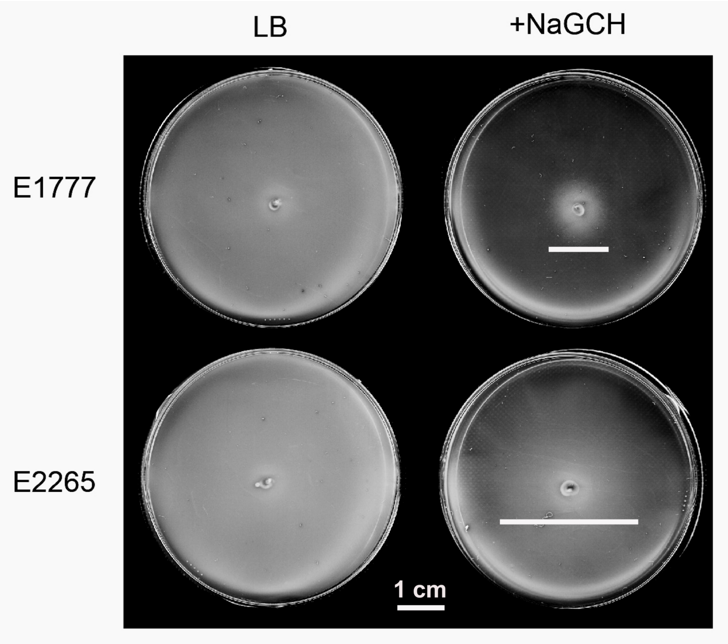

Figure S2. Influence of NaGCH on bacterial motility. ETEC strains E1777 and E2265 were inoculated into swimming plates with and without 0.2% NaGCH and the motility diameter (white lines) was measured after 8 hours of growth at 37°C. Representative images are shown.

TABLES

Table S1. List of genes differentially expressed in the presence of bile or NaGCH in ETEC E1777 or E2265.

Table S2. List of proteins differentially produced in the presence of bile or NaGCH in ETEC E1777 or E2265.

Table S3. Strains, plasmids, and primers used in this study.
